# Prognostic value of auditory evoked potentials in disorders of consciousness: a systematic literature review

**DOI:** 10.1016/j.cnp.2026.01.005

**Published:** 2026-01-30

**Authors:** Panithi Khusakul, Ioan Valnarov-Boulter, Aden Noronha, Wanapas Wachiradejkul, Tim M. Young, Blake Hale

**Affiliations:** a University College London Medical School, Gower Street, London WC1E 6BT, UK; b Princess Srisavangavadhana Faculty of Medicine, Chulabhorn Royal Academy, Bangkok 10210, Thailand; c Queen Square Institute of Neurology, University College London, London WC1N 3BG, UK; d Department of Clinical Neurophysiology, The National Hospital for Neurology & Neurosurgery, University College London Hospital, 23 Queens Square, London WC1N 3BG, UK

**Keywords:** Auditory evoked potential, Disorder of consciousness, Mismatch negativity, Brainstem auditory evoked potentials, Coma, Prognosis

## Abstract

**Objective:**

Decisions regarding withdrawal of life support in disorders of consciousness (DoC) are challenged by prognostic uncertainty. Auditory evoked potentials (AEPs) are widely used, yet no recent systematic review has focused exclusively on their prognostic value. Given evidence that some withdrawal decisions are based on inaccurate prognoses, this study aimed to evaluate the prognostic utility of AEPs in DoC using recent evidence.

**Methods:**

Comprehensive literature searches of PubMed, Scopus, and Web of Science Core Collection identified studies published between 2013 and 2023 assessing AEPs for prognostication in DoC. Three authors independently screened studies using predefined criteria. Eligible studies were original English-language research evaluating prognostic outcomes in adults (≥18 years) with DoC. Reviews and secondary literature were excluded. Risk of bias was assessed using the QUADAS-2 tool.

**Results:**

Of 2249 records screened, 24 studies met inclusion criteria, encompassing 1562 patients. Mismatch negativity (MMN), the most frequently studied AEP, reliably predicted both favourable and unfavourable outcomes. P300 demonstrated potential for predicting recovery, though findings were inconsistent. Although less studied, the absence of N100 predicted poor functional outcomes, including reduced autonomy. Absence of brainstem or middle-latency AEPs predicted negative outcomes with high specificity but low sensitivity.

**Conclusion:**

MMN, P300, and N100 demonstrate meaningful prognostic value in DoC. Despite methodological heterogeneity, current evidence supports AEPs as useful clinical prognostic tools. Standardised, blinded protocols are needed to improve reliability.

**Significance:**

Integrating AEPs into multimodal prognostic frameworks may enhance outcome prediction. Advances in electrode coverage, machine learning, and automated detection paradigms offer promising future directions.

## Highlights

• Auditory evoked potentials can be used for prognosis in disorders of consciousness.
• Mismatch negativity predicts outcomes in consciousness disorders, literature shows.
• Absence of brainstem or middle-latency auditory potentials predicts poor outcomes.

## 1 Introduction

### 1.1 Background – disorders of consciousness

Disorders of Consciousness (DoC) encompass conditions with impaired consciousness, with major causes including traumatic brain injury (TBI) and brain anoxia from cardiac arrest (Edlow et al., 2021). The three main categories of DoC are coma, unresponsive wakefulness syndrome (UWS), and minimally conscious state (MCS) (Giacino et al., 2014). Coma is characterised by the complete loss of both arousal and awareness, presenting with continuously closed eyes, absent sleep-wake cycles and motor activity limited to primitive reflexes (Giacino et al., 2014, Huff and Tadi, 2020). UWS, formerly termed Vegetative State, is characterised by the recovery of arousal with spontaneous eye opening and intermittent wakefulness, but with the complete absence of language comprehension, communication, or reproducible purposeful behaviour (Giacino et al., 2014, Gosseries et al., 2011, van Erp et al., 2014). MCS is distinguished by inconsistent yet clearly discernible behavioural evidence of self or environmental awareness. Diagnosis of MCS requires the demonstration of at least one reproducible sign of volition, such as simple command-following, intelligible verbalisation, or non-reflexive behaviours like sustained visual pursuit (Cranford, 2002, Giacino et al., 2014. MCS positive and MCS negative groups can be established based on the presence of command-following, gestural or verbal communication (Thibaut et al., 2019). To reinforce accuracy of classification and mitigate diagnostic error, the 2018 Practice Guideline Update (Giacino et al., 2018) mandates the use of standardised measures such as the Coma-Recovery Scale-Revised (CSR-R). Furthermore, the guideline recommends serial assessments to account for fluctuations in responsiveness and the identification of confounding conditions prior to diagnosis.

### 1.2 Current methods for prognostication in disorders of consciousness patients

Currently, a core element in DOC prognostication is the neurological examination (Edlow et al., 2021). Absence of pupillary and corneal reflexes by day three after onset of DoC has been associated with unfavourable outcomes (Greer, 2015). However, these predictors can have multiple confounding variables, such as the patient’s medication, hypothermia treatment, and examiner variability (Edlow et al., 2021). Existing clinical scoring systems, such as the Glasgow Coma Scale (GCS) and Glasgow Outcome Scale (GOS), are commonly used in DoC assessment but are constrained by subjectivity and limitations in capturing subtle changes in consciousness (Rittenberger et al., 2011, McMillan et al., 2016). Serum biomarkers such as neuron-specific enolase and S-100B have been used for coma prognostication but are limited by inconsistent thresholds and extra-neuronal sources (Sandroni et al., 2014). Brain imaging, primarily with CT scans, detects mortality indicators like global oedema in cardiac arrest and subarachnoid haemorrhage (Edlow et al., 2021), but has limited sensitivity for coma causes. Magnetic resonance imaging (MRI) of the brain is more sensitive to coma-causing lesions; however, these findings are only seen in a subpopulation of DoC patients. (Edlow et al., 2021). Whilst electroencephalograms (EEGs) can contribute to the prognostication of comatose patients (Guedes et al., 2020, Westhall et al., 2016), their application to other DoC categories remains relatively limited (Azabou et al., 2018, Comanducci et al., 2020). Somatosensory evoked potentials (SSEPs), particularly the bilateral absence of the N20 wave, have demonstrated reliable prognostic value in predicting unfavourable outcomes in DoC patients (Kromm et al., 2023, Sandroni et al., 2014). However, the binary presence of N20 potentials has traditionally been considered insufficient to guarantee a favourable outcome, although emerging evidence suggests that quantitative amplitude analysis may improve predictive accuracy for recovery. (Comanducci et al., 2020, Kromm et al., 2023).

### 1.3 Auditory evoked potentials

Auditory evoked potentials (AEPs) are generated in response to acoustic stimuli delivered through earphones (American Clinical Neurophysiology Society (ACNS), 2006, Plourde, 2006). These time-locked signals are captured using head electrodes, averaged from the ongoing EEG, and then analysed (American Clinical Neurophysiology Society (ACNS), 2006, Duncan et al., 2009). AEPs can be divided into brainstem, middle-latency, and long-latency potentials (BAEPs, MLAEPs, and LLAEPs, respectively) (Duncan et al., 2009). While shorter latency responses evidence stimulation detection, successive waveforms can reveal signs of consciousness in patients and evaluate their capacity to interact with their environment. Existing evidence suggests that present LLAEPs (mismatch negativity (MMN) and P300) may be effective predictors of awakening in patients in a coma or similar conditions (Comanducci et al., 2020, Daltrozzo et al., 2007).

### 1.4 Rationale for study

In patients who are comatose post-cardiac arrest, 40–60% of deaths occur after a decision to withdraw life-sustaining therapy due to a predicted poor neurological prognosis (Henson et al., 2021, Turner-Stokes et al., 2022). However, nearly 20% of such cases might have been inaccurately given an unfavourable prognosis, leading to potentially premature decisions about withdrawing life-sustaining therapy (Henson et al., 2021). This highlights the need for a more accurate method of prognostication. The current American Academy of Neurology practice guidelines on DoC date from 2018 and do not specifically discuss AEPs (Giacino et al., 2018). A number of relevant investigative studies on the prognostic value of AEPs on DoC patients have been published since then, supporting the need for our systematic review.

## 2 Aims

This study aims to comprehensively search for, and analyse, available evidence published in the last decade on the use of AEPs in prognosticating patients with DoC.

## 3 Methods

### 3.1 Study design and search strategy

The protocol for this systematic review was not registered in a public registry because it was initiated as a university degree dissertation. This study adheres to the Preferred Reporting Items for Systematic Reviews and Meta-Analyses (PRISMA) 2020 guidelines; the completed PRISMA checklist is available in Supplementary File 1.

A comprehensive literature search was conducted using three different databases: PubMed, Scopus, and Web of Science Core Collection. The search terms applied to both titles and abstracts in all three databases included: ('auditory evoked potential' OR 'auditory event-related potential' OR 'brainstem auditory evoked potential' OR 'BAEP' OR 'mismatch negativity' OR 'middle latency auditory evoked potential' OR 'MLAEP' OR 'long latency event-related potential' OR 'ERP') AND ('prognostication' OR 'prognosis'). The search strategy was designed to maximise sensitivity to ensure no relevant studies on AEPs in disorders of consciousness were missed, while maintaining sufficient specificity. A filter from the last decade (2013–2023) was applied to ensure a focus on current evidence. The last search was conducted on December 29, 2023.

### 3.2 Eligibility criteria

During the screening process, the inclusion and exclusion criteria (Table 1) were applied to identified records. Only articles including patients aged 18 years or older were included, as paediatric cohorts may have differing prognostication.

**Table 1:** Inclusion and exclusion criteria for the selection of articles.

*Inclusion Criteria*
1. The study explored at least one AEP.
2. The study investigated patients with at least one type of DoC, or that the majority of the participants had this diagnosis.
3. The study examined the capacity of AEPs to prognosticate patients with DoC.
4. Patients included in the study were aged 18 years or older.

*Exclusion Criteria*
1. The primary focus of the study was not the prognostic potential of AEPs.
2. Full-text articles not available in English
3. The article was in the format of a review, letter, opinion piece, case study, or survey.

Abbreviations: AEP – Auditory Evoked Potential; DoC – Disorder of Consciousness.

### 3.3 Study selection

Duplicate articles were identified and removed using EndNote’s automation tool, with manual verification. The selection of articles was independently performed by three co-authors (IV-B, AN, WW), covering both title/abstract screening and full-text review. Any disagreements were resolved through consensus. EndNote and Rayyan were used to screen articles and facilitate decision-making on articles through full-text screening.

### 3.4 Quality assessment

The methodological quality of the included studies was assessed using the Quality Assessment of Diagnostic Accuracy Studies-2 (QUADAS-2) tool (Whiting et al., 2011). The QUADAS-2 tool evaluates risk of bias and applicability concerns across four domains: Patient Selection, Index Test, Reference Standard, and Flow and Timing. Two reviewers independently evaluated each study. Risk of bias was judged as low, high, or unclear/no information based on the signalling questions in each domain. Disagreements between reviewers were resolved through discussion and consensus. The results of the assessment were illustrated in a traffic-light plot generated using the Robvis online tool (McGuinness and Higgins, 2021).

### 3.5 Data extraction and synthesis

Prognostic data were extracted from each study and grouped for synthesis according to the AEP modality used. This process was carried out independently by four co-authors (PK, IV-B, AN, WW), with any disagreements resolved through discussion and consensus. Data were tabulated to display study characteristics, patient demographics, and prognostic findings. Due to the considerable heterogeneity in methodologies and results across the studies included in this systematic literature review, a *meta*-analysis was not possible. Tables were constructed to support a narrative synthesis of prognostic findings and a qualitative exploration of heterogeneity factors, such as sedation protocols and duration between DoC onset and recording. For studies reporting sufficient raw data to construct 2 × 2 contingency tables (number of patients with positive versus negative AEP responses cross-tabulated by favourable versus poor outcomes), sensitivity and specificity with 95% confidence intervals were calculated in Stata. In studies where sensitivity and specificity values were already reported by the authors, these were extracted directly. Sensitivity analyses were not performed due to the lack of quantitative synthesis.

## 4 Results

### 4.1 Study selection

**Fig. 1:**
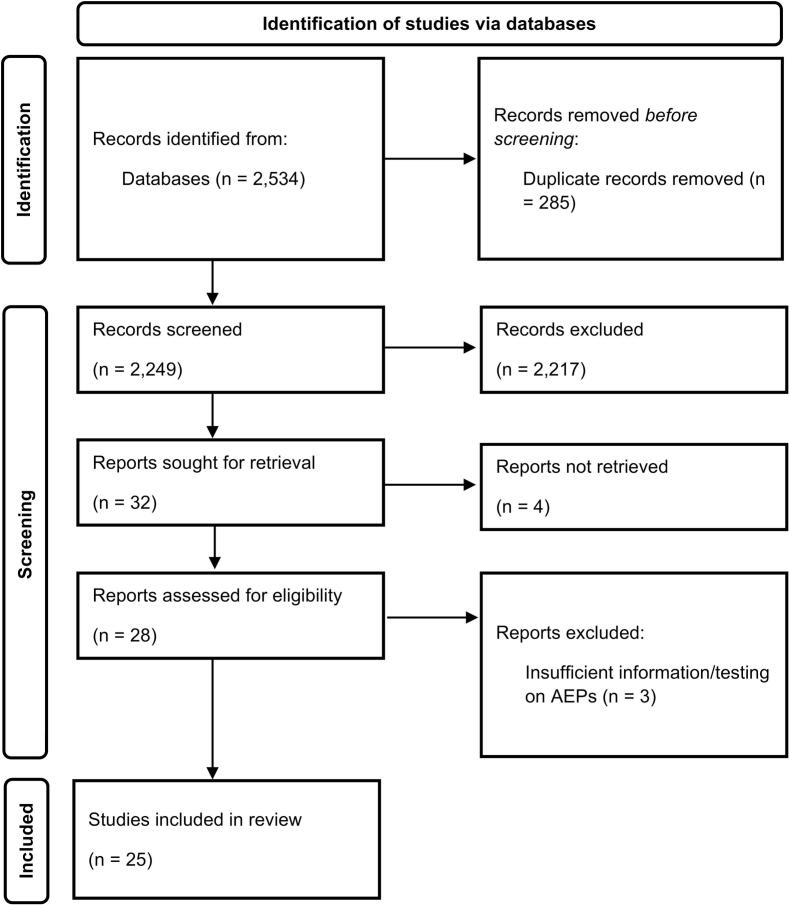
PRISMA Flowchart Displaying the Study Search and Selection Process. This flowchart adapted based on the “PRISMA, 2020 flow diagram for new systematic reviews which included searches of databases and registers only” (PRISMA, 2020), illustrates the study selection process. Of the 2534 articles identified, 285 duplicates were removed, and 2217 were excluded based on the inclusion and exclusion criteria. Thirty-two articles were sought for retrieval, four were unavailable, and four were excluded upon full-text review for insufficient AEP focus. Twenty-four articles were ultimately included in this study. Abbreviation: AEP – auditory evoked potential Note: Fig. 1 does not require colour and can be represented in grayscale.

A PRISMA flow diagram is shown in Fig. 1, showing the process of study selection. The initial search in PubMed, Scopus, and Web of Science yielded a total of 2534 records (2121, 260, and 153 results, respectively). After removing 285 duplicate articles, 2249 articles were left to be screened. The majority of articles were excluded because the primary focus of the article did not concern the prognostic potential of AEPs. Upon review, 32 articles met the specified inclusion criteria, of which, four articles could not be retrieved. Therefore 28 studies were assessed for eligibility and reviewed in full by four co-authors (PK, IV-B, AN, WW). Out of the 28 full-text articles assessed for eligibility, four were excluded due to ineligible index tests or insufficient data. A list of excluded studies at this stage with reasons are provided in Supplementary Table S1. A final number of 24 articles were included in this study.

### 4.2 Study and patient characteristics

**Fig. 2:**
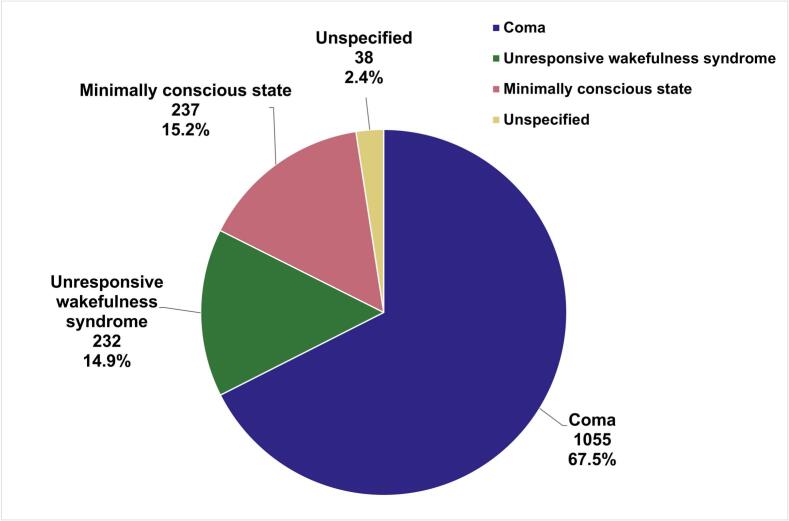
Distribution of Disorder of Consciousness Types in 1562 Patients Across 24 Studies. This pie chart depicts the proportions of different types of disorders of consciousness identified among the reviewed articles. Coma was the most frequently reported type (1055 cases, 67.5%), followed by minimally conscious state (237 cases, 15.2%), and unresponsive wakefulness syndrome (232 cases, 14.9%). A small proportion of cases were unspecified in the study (38 cases, 2.4%) Abbreviations: MCS – minimally conscious state; UWS – unresponsive wakefulness syndrome Note: Colour is essential for an accurate interpretation of Fig. 2.

**Fig. 3:**
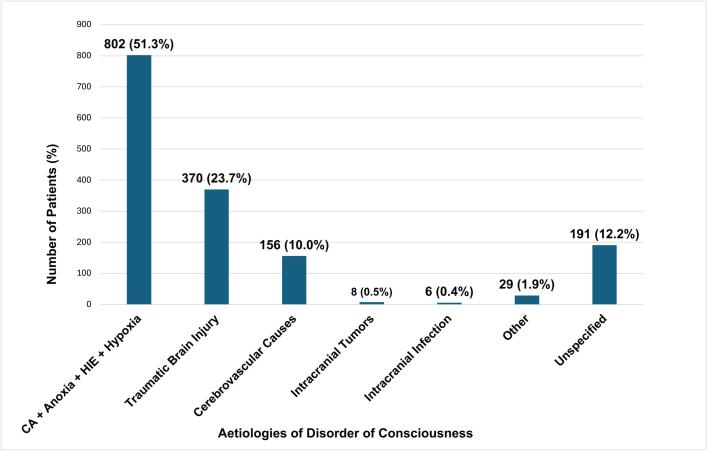
Aetiologies of Disorders of Consciousness in 1562 Patients Across 24 Studies. Distribution of aetiologies contributing to disorders of consciousness, highlighting the predominant causes among included articles. A combination of cardiac arrest, anoxia, hypoxic-ischemic encephalopathy and hypoxia were the most common (802 cases, 51.3%), followed by traumatic brain injury (370 cases, 23.7%), and cerebrovascular cases (156 cases, 10.0%). Less frequently reported aetiologies include intracranial tumours (8 cases, 0.5%), intracranial infections (6 cases, 0.4%), other causes (29 cases, 1.9%) and unspecified aetiologies (191 cases, 12.2%). This distribution underscores the diverse mechanisms underlying DoC in our study while highlighting the predominance of cardiac arrest-related aetiologies. Abbreviations: DoC – Disorder of Consciousness; CA – cardiac arrest; HIE – hypoxic-ischemic encephalopathy **Note:** Colour is essential for an accurate interpretation of Fig. 3.

Out of the 24 included studies, 13 were prospective studies, while 11 were retrospective. The cumulative sample comprised 1562 DoC patients. The most common type of DoC among the patients was coma, making up 67.5% (1055) of patients (Fig. 2), followed by MCS and UWS. The leading aetiologies for DoC were one or more of cardiac arrest, anoxia, hypoxia, and hypoxic-ischemic encephalopathy (HIE), as shown in Fig. 3. These aetiologies were combined in our study due to their frequent co-occurrence, as the majority of anoxia, hypoxia and HIE cases reported across the articles were secondary to cardiac arrest. Regarding index tests, MMN was most frequently evaluated (13 studies), followed by P300 (6 studies) and BAEP (4 studies). MLAEPs were assessed in two studies, N100 in three studies, and N400 in two studies. Table 2 summarises the types of AEPs, number of patients, types and aetiologies of DoC and main prognostic findings. Study characteristics, including country of origin, study type, and patient demographics (specifically mean age and sex distribution) are provided in Supplementary Table S2. Methodological data, such as sedation protocols, measure of outcomes and interval from DoC onset to AEP recording in each study are provided in Supplementary Table S3.

**Table 2:** Type of auditory evoked potentials investigated, number of patients, type of doc, aetiologies and key findings from each included study.

Author & year	AEP modalities studied	Patients, type of DoC and aetiologies	Main findings related to AEPs in DoC Prognostication
1. Chen et al., 2020	40-Hz Auditory Steady-State Response	*Patients:* 32 DoC patients – Coma (n = 32) *Aetiologies:* TBI (n = 13), stroke (n = 14), cardiac arrest (n = 3), Others (n = 2)	– The absence of 40-Hz Auditory Steady-State Response in multiple stimulation paradigms was generally associated with an unfavourable prognosis.
2. Floyrac et al., 2023	MMN	*Patients:* 29 DoC patients – Coma (n = 29) *Aetiologies:* cardiac arrest (n = 29)	– A machine learning approach to analyse AEPs revealed the limited effectiveness of MMN presence in identifying patients with good neurological outcomes.
3. Gobert et al., 2018	BAEP	*Patients:* 7 DoC patients – Coma (n = 7) *Aetiologies:* Subarachnoid haemorrhage (n = 7)	– Alterations or absence of BAEP in TBI patients were associated with the absence of recovery.
4. Jaeger et al., 2014	MLAEP N100 MMN P300	*Patients:* 18 DoC patients – Coma (n = 18) *Aetiologies:* TBI (n = 18)	– MLAEP abolition was reported to predict unfavourable outcomes but with low sensitivity. – Absence of N100 was significantly associated with lower autonomy, measured by the Functional Independence Measure (r = 2.3, p = 0.022).
5. Juan et al., 2016	Auditory discrimination using MMN	*Patients:* 32 DoC patients – Coma (n = 32) *Aetiologies:* Anoxia (n = 32)	– Auditory discrimination progression was positively correlated with better cognitive performances and negatively correlated with coma duration (p = 0.02) and hospital stay duration (p = 0.005).
6. Levi-Strauss et al., 2023	MMN P300	*Patients:* 38 DoC patients – Unspecified DoC *Aetiologies:* cardiac arrest (n = 38)	– A statistically significant trend was observed with GOSE scores (p = 0.003) and mortality at three months (p = 0.03) using a combination of presence/absence of MMN and P300.
7. Lim et al., 2021	BAEP P100	*Patients:* 185 DoC patients – Coma (n = 185) *Aetiologies:* cardiac arrest (n = 185)	– The absence BAEPs had a limited sensitivity of 9.4% (95% CI = 4.6–16.7%) in predicting unfavourable neurological outcomes at six months, making them less effective predictors for coma emergence.
8. Liu et al., 2021	MMN	*Patients:* 113 DoC patients – Coma (n = 113) *Aetiologies:* Stroke (n = 65), HIE from cardiac arrest (n = 28), intracranial infection (n = 6), unspecified (n = 14)	– N60 and MMN were strong in prognosticating awakening in patients, and their combination further improved prediction.
9. Meiron et al., 2021	MMN	*Patients:* 10 DoC patients – UWS (n = 9), MCS (n = 1) *Aetiology:* Anoxia (n = 6), others (n = 4)	– DoC survivors showed significantly smaller baseline MMN compared to non-survivors, who demonstrated paroxysmal and 80% larger MMN amplitudes which could predict clinical outcomes in DoC patients over a year. – Baseline MMN latency showed the ability to predict change in GCS scores at 1-year post-baseline assessment
10. Morgalla and Tatagiba, 2014	BAEPs	*Patients:* 100 DoC patients – Coma (n = 100) *Aetiologies:* TBI (n = 100)	– No single parameter, including BAEP wave I-V interpeak latency and V/I amplitude ratio, could reliably predict long-term clinical outcomes from coma patients after severe traumatic brain injury.
11. Morlet et al., 2023	Paradigm using P300 and N200	*Patients:* 68 DoC patients – Coma (n = 37), UWS (n = 17), MCS (n = 14) *Aetiologies:* Anoxia (n = 19), brain trauma (n = 22), stroke (n = 22), others (n = 5)	– A paradigm using a of P300 and N200 was able to predict awakening with a low sensitivity (32%) and a high specificity of (96%). – There was a strong correlation between the active test in awakening patients and functional dependency after six months.
12. Obinata et al., 2020	BAEP	*Patients:* 124 DoC patients – Coma (n = 124) *Aetiologies:* cardiac arrest (n = 124)	– Absence of BAEP wave V is highly predictive of unfavourable neurological outcomes.
13. Perez et al., 2021	Auditory Event-Related ‘Global Effect’	*Patients:* 309 DoC patients – UWS (n = 138), MCS (n = 171) *Aetiologies:* TBI (n = 70), Anoxia (n = 101), Others (stroke, hematoma, encephalitis, or toxic encephalopathy) (n = 138)	– Global effect in brain activity was a strong indicator of potential recovery of consciousness among patients with consciousness disorders. – Initial positivity of the Global effect was able to predict 6-month recovery of consciousness
14. Pfeiffer et al., 2017	Auditory discrimination using MMN	*Patients:* 60 DoC patients – Coma (n = 60) *Aetiology:* Cardiac arrest (n = 60)	– Tracking auditory discrimination can help predict good recovery regardless of the temperature target.
15. Portnova et al., 2023	P100 N100 P200 N200 P300 N400	*Patients:* 24 DoC patients – Coma (n = 24) *Aetiology:* TBI (n = 24)	– P200 and N200 amplitudes using emotional stimuli significantly correlated with GOSE scores, highlighting their potential as biomarkers for recovery of consciousness (P200: r = 0.6, p 0.0014; N200: r = -0.56, p 0.0037).
16. Rodriguez et al., 2014	BAEPs MLAEPs (Pa wave) N100 MMN	*Patients:* 17 DoC patients – Coma (n = 17) *Aetiologies: C*ardiac arrest (n = 12), other causes (n = 5)	– Presence of BAEPs (bilaterally) and MLAEP Pa waves were highly predictive of patient – The presence of MMN during coma was a strong predictor of patient awakening – MMN was shown to be the best predictor of patient awakening, and more reliable than BAEPs and MLAEPs.
17. Rossetti et al., 2014	Auditory discrimination using MMN	*Patients:* 30 DoC patients treated with therapeutic hypothermia – Coma (n = 30) *Aetiologies:* HIE (n = 30)	– Improvement in auditory discrimination predicted awakening with high specificity – The addition of the Automated Auditory Discrimination Paradigm based on MMN to standard prognostic tools greatly enhanced the prediction of awakening in coma patients post-cardiac arrest with therapeutic hypothermia.
18. Steppacher et al., 2013	P300 N400	*Patients:* 92 DoC patients – UWS (n = 53), MCS (n = 39) *Aetiologies:* TBI = 43, hypoxia = 25, others = 24	– The presence of N400 was able to strongly predict favourable recovery outcomes in patients with DoC. – P300 did not show a significant predictive relationship with patient outcomes.
19. Tzovara et al., 2013	Auditory discrimination using MMN	*Patients:* 30 DoC patients – Coma (n = 30) *Aetiologies:* cardiac arrest (n = 30)	– Change in auditory discrimination from therapeutic hypothermia to normothermic state was able to predict clinical outcomes with 70% accuracy – Improvement in auditory discrimination using MMN predicted awakening and survival at 3 months with a high positive predictive value
20. Tzovara et al., 2016	Auditory discrimination using MMN	*Patients:* 94 DoC patients – Coma (n = 94) *Aetiologies:* cardiac arrest (n = 75), unspecified (n = 3), other (n = 16)	– Improvement in auditory discrimination from hypothermia to normothermia was highly predictive of patients’ emergence from coma.
21. Wang et al., 2017	MMN P300	*Patients:* 11 DoC patients – UWS (n = 6), MCS (n = 5) *Aetiologies:* TBI (n = 2), Cerebral Infarction (n = 1), HIE (n = 2), Brainstem Infarction (n = 1), Encephalorrhagia (n = 5)	– Demonstrated limited value for P300 and MMN responses for the prediction of recovery of consciousness.
22. Wang et al., 2022	MMN P300	*Patients:* 68 DoC patients – Unspecified DoC *Aetiologies:* TBI (n = 47), Subarachnoid haemorrhage (n = 8), Intracerebral haemorrhage (n = 8), Others (n = 5)	– DoC patients demonstrated better 6-month neurological outcomes with a shorter P3a latency (p = 0.044), larger P3a amplitude (p < 0.001), and large MMN amplitude (p = 0.001). – Both the MMN amplitude and the P3a amplitude were significant independent predictors of a favourable outcome.
23. Zhang et al., 2017	P300	*Patients:* 18 DoC patients – Coma (n = 2), UWS (n = 9), MCS (n = 7) *Aetiologies:* Anoxia (n = 3), intracranial haemorrhage (n = 7), TBI (n = 8)	– Absence of P300 from both paradigms using the subject’s own name and standard stimuli predicted poor recovery with high sensitivity and specificity
24. Zhou et al., 2021	MMN	*Patients:* 53 DoC patients – Coma (n = 53) *Aetiologies:* TBI (n = 22), cerebrovascular diseases (n = 16), intracranial tumours (n = 5), unspecified (n = 10)	– Supported use of MMN amplitude as a prognostic indicator for comatose patients with severe brain injury.

Abbreviations: AEP – Auditory Evoked Potential; BAEP – Brainstem Auditory Evoked Potential; CI – Confidence Interval; DoC – Disorder of Consciousness; EEG – Electroencephalogram; GCS – Glasgow Coma Scale; GOSE – Glasgow Outcome Scale-Extended; HIE – Hypoxic-Ischemic Encephalopathy; MCS – Minimally Conscious State; MLAEP – Middle-Latency Auditory Evoked Potential; MMN – Mismatch Negativity; NPV – Negative Predictive Value; PPV – Positive Predictive Value; SAH – Subarachnoid Haemorrhage; SD – Standard Deviation; TBI – Traumatic Brain Injury; UWS – Unresponsive Wakefulness Syndrome.

### 4.3 Risk of bias assessment

**Fig. 4:**
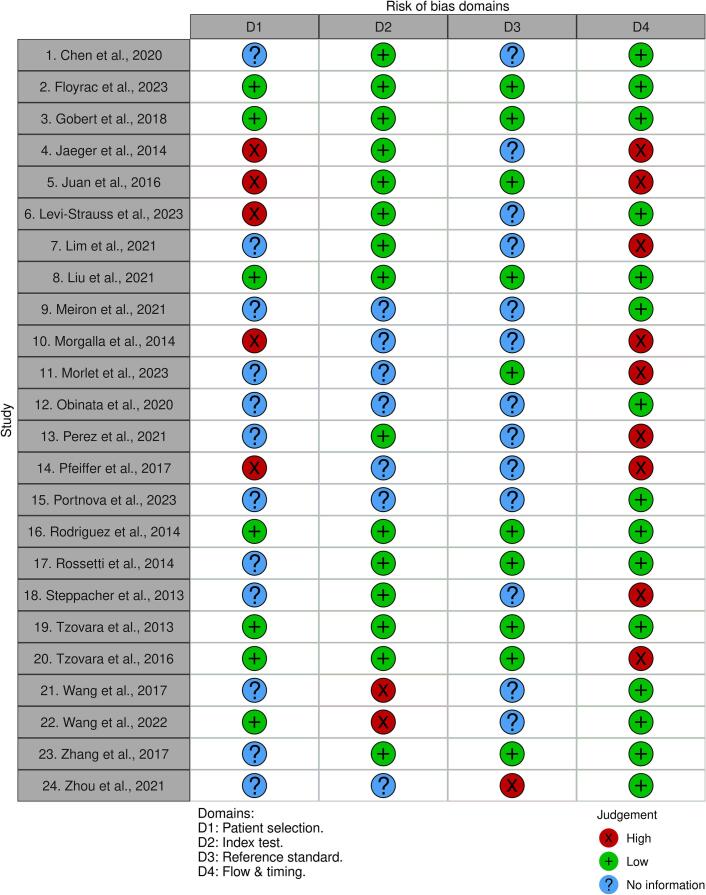
Risk of Bias Assessment for Included Studies using QUADAS-2. This traffic light plot illustrates the domain-level judgments for each of the 24 included studies in this review based off the QUADAS-2 Risk of Bias Judgment Tool. The assessment covers four key domains: Patient selection, Index test, Reference standard, and Flow and timing. Green circles indicate a low risk of bias, red circles indicate a high risk of bias, and blue circles indicate unclear risk due to insufficient information. Note: Colour is essential for an accurate interpretation of Fig. 4.

The risk of bias assessment (QUADAS-2) is summarised in Fig. 4, with detailed domain judgments and applicability concerns provided in Supplementary Table S4. The Patient Selection domain presented reporting gaps, where 12 studies lacked clear recruitment protocols. While the Index Test domain was generally low risk, two studies were rated high risk due to the lack of pre-specified thresholds (Wang et al., 2017, Wang et al., 2022). In the Reference Standard domain, blinding of outcome assessors was frequently unreported (14 studies). The Flow and Timing domain exhibited the highest prevalence of bias, as nine studies showing significant attrition or the exclusion of patients from the final analysis.

### 4.4 Overview of prognostic data

Quantitative prognostic data were synthesised where available. In cases where the sensitivity and specificity were available or could be calculated, these data are summarised in Table 3.

**Table 3:** Sensitivity and specificity of auditory evoked potentials in predicting favourable outcomes in patients with disorders of consciousness across studies.

Study	Number of patients	Type of DoC	Measure of ‘favourable’ outcome	Sensitivity (95% CI)	Specificity (95% CI)
*Brainstem Auditory-Evoked Potential (Presence)*					
Gobert et al., 2018	7	Coma (n = 7)	**[Short-Term]** EEG Reactivity	1.00 (0.03, 1.00)	0.50 (0.12, 0.88)
Lim et al., 2021	185	Coma (n = 185)	**[Long-term]** CPC < 3 at 6 months	1.00 (0.88, 1.00)	0.09 (0.05, 0.17)
Obinata et al., 2020	124	Coma (n = 124)	**[Short-Term]** CPC < 3 at discharge	1.00 (0.79, 1.00)	0.44 (0.34, 0.53)

*P100*					
Lim et al., 2021	186	Coma (n = 185)	**[Long-term]** CPC 1–2 at 6 months	1.00 (0.88, 1.00)	0.54 (0.46, 0.63)

*Auditory Steady-State Response*					
Chen et al., 2020	32	Coma (n = 32)	**[Long-term]** GOS ≥ 3 at 6 months	1.00 (0.59, 1.00)	0.76 (0.55, 0.91)

*Mismatch Negativity (Presence)*					
Floyrac et al., 2023	29	Coma (n = 29)	**[Short-Term]** Command-following at discharge	0.33*	0.82*
Levi-Strauss et al., 2023	38	Unspecified	**[Long −term]** GOSE ≥ 3 at 3 months	0.67 (0.22, 0.96)	0.90 (0.73, 0.98)
Liu et al., 2021	113	Coma (n = 113)	**[Long −term]** GOS ≥ 3 at 3 months	0.62*	0.82*
Wang et al., 2017	11	UWS (n = 6) MCS (n = 5)	**[Short-term]** Clinical Improvement (UWS → MCS)	1.00 (0.40, 1.00)	0.14 (0.00, 0.58)

*Mismatch Negativity (Improvement in Auditory Discrimination)*					
Juan et al., 2016	32	Coma (n = 32)	**[Long-term]** CPC < 2 at 3 months	0.67 (0.41, 0.87)	0.86 (0.57, 0.98)
Pfeiffer et al., 2017	60	Coma (n = 60)	**[Long-term]** CPC 1–3 within 3 months	0.50 (0.32, 0.68)	0.69 (0.48, 0.86)
Rossetti et al., 2014	30	Coma (n = 30)	**[Long-term]** CPC < 3 at 3 months	0.56 (0.31, 0.78)	1.00 (0.74, 1.00)
Tzovara et al., 2016	94	Coma (n = 94)	**[Long-term]** Survival at 3 months	0.48 (0.35–0.62)	0.84 (0.68–0.94)

*Paradigm using P300 and N200*					
Morlet et al., 2023	68	Coma (n = 37) UWS (n = 17) MCS (n = 14)	**[Long-term]** GOS-E 3–5 at 6 months	0.32*	0.96*

*P300 (Presence)*					
Levi-Strauss et al., 2023	38	Unspecified	**[Long-term]** GOSE ≥ 3 at 3 months	0.83 (0.36, 1.00)	0.66 (0.46, 0.82)
Steppacher et al., 2013	92	UWS (n = 53) MCS (n = 39)	**[Long-term]** Recovery of consciousness (Functional Communication)	0.34 (0.18, 0.54)	0.81 (0.67, 0.90)
Wang et al., 2017	11	UWS (n = 6) MCS (n = 5)	**[Short-Term]** Clinical Improvement (UWS → MCS)	0.75 (0.19, 0.99)	0.14 (0.00, 0.58)
Zhang et al., 2017	18	Coma (n = 2) UWS (n = 9) MCS (n = 7)	**[Long-term]** Clinical Improvement (UWS → MCS) at 12 months	0.80 (0.28, 0.99)	0.92 (0.64, 1.00)
Auditory Event-Related Global Effect					
Perez et al., 2021	309	Coma (n = 138) MCS (n = 171)	**[Long-term]** Consciousness Recovery at 6 months	0.35 (0.25, 0.45)	0.84 (0.71, 0.93)
N400 (Presence)					
Steppacher et al., 2013	92	UWS (n = 53) MCS (n = 39)	**[Long-term]** Recovery of Consciousness (Functional Communication)	0.34 (0.18, 0.54)	0.81 (0.67, 0.90)

^1^Abbreviations: CI – Confidence Interval; CPC – Cerebral Performance Category; DoC – Disorder of Consciousness; EEG – Electroencephalography; GOS – Glasgow Outcome Scale; GOSE – Glasgow Outcome Scale-Extended; MCS – Minimally Conscious State; UWS – Unresponsive Wakefulness Syndrome.

*95% CI not reported in the original study and raw data unavailable for calculation

## 5 Discussion

This systematic literature review presents available evidence from the last decade on the value of AEPs in prognosticating patients with DoC. Of the evaluated potentials, MMN yielded the most extensive evidence base, and emerged as a strong predictor of both positive and negative outcomes in DoC. Meanwhile, P300 showed potential but was limited by conflicting evidence (Jaeger et al., 2014, Liu et al., 2021, Rodriguez et al., 2014, Wang et al., 2022, Zhou et al., 2021, Floyrac et al., 2023, Juan et al., 2016; Levi-Strauss et al., 2023; Meiron et al., 2021, Rodriguez et al., 2014, Tzovara et al., 2016). The absence of BAEPs exhibited high specificity in prognosticating poor outcomes but was constrained by low sensitivity (Gobert et al., 2018, Lim et al., 2021, Obinata et al., 2020; Rodriguez et al., 2014). Finally, the utility of MLAEPs, N100 and N400 remains underexplored, requiring further research (Jaeger et al., 2014, Portnova et al., 2023, Rodriguez et al., 2014, Steppacher et al., 2013). Factors such as sedation use and variability in the timing of AEP testing add challenges to the interpretation of the prognostic utility of these AEPs.

### 5.1 Long-latency auditory evoked potentials

#### 5.1.1 Mismatch negativity

##### 5.1.1.1 Prognostic value of mismatch negativity presence

MMN represents activity in the auditory and frontal cortices when acoustic deviants are mismatched to the memory trace of the ongoing standard stimuli, even when patients appear to be inattentive to the changes (Näätänen et al., 1978, Duncan et al., 2009). The presence of MMN and larger MMN amplitudes have shown significant predictive power for favourable neurological outcomes for DoC patients. (Jaeger et al., 2014, Liu et al., 2021, Rodriguez et al., 2014, Wang et al., 2022, Zhou et al., 2021). Wang and colleagues demonstrated a strong correlation between greater MMN amplitudes and positive neurological outcomes at six months (Wang et al., 2022). Improvement in auditory discrimination utilising MMN have demonstrated strong predictive value for positive outcomes in patients with DoC (Juan et al., 2016, Pfeiffer et al., 2017; Rossetti et al., 2014; Tzovara et al., 2013, Tzovara et al., 2016). In addition, Zhou and colleagues showed that MMN amplitudes were able to predict the awakening of patients with a sensitivity of 81.1% and specificity of 68.7% (Zhou et al., 2021). However, limitations such as the inclusion of patients receiving sedatives without stratification based on the use of these medications, reduce the generalisability of these results.

The findings for the prognostic value of MMN in our study corroborate an earlier review (Pruvost-Robieux et al., 2022), suggesting that the recording of an MMN is a robust predictive marker of awakening, especially in post-anoxic DoC patients. Whilst the review by Pruvost-Robieux and colleagues focused on MMN as a predictive marker of awakening, our study also incorporated articles which specifically assessed MMN’s ability to predict negative outcomes (Floyrac et al., 2023, Juan et al., 2016; Levi-Strauss et al., 2023; Pruvost-Robieux et al., 2022, Rodriguez et al., 2014, Tzovara et al., 2016). The consistency across multiple studies suggests that the presence of MMN could serve as a cornerstone for prognostic evaluation in the population of DoC patients.

##### 5.1.1.2 Prognostic value of mismatch negativity absence

The absence of MMN has evidence as a predictor of negative outcomes in DoC patients (Floyrac et al., 2023, Juan et al., 2016; Levi-Strauss et al., 2023; Meiron et al., 2021, Rodriguez et al., 2014, Tzovara et al., 2016). Levi-Strauss and colleagues (2023) analysed a cohort of 38 DoC patients, showing that 19 out of 28 patients who exhibited a Glasgow Coma Outcome Scale Extended score of 1–2 (death or persistent UWS) after three months, lacked both MMN and P300. Rodriguez and colleagues further showed that none of the three patients with absent MMN in a cohort of 17 comatose patients, awakened. These findings suggest that the absence of MMN has utility as a negative prognostic marker. However, methodological constraints such as small sample sizes and the long interval from DoC onset to AEP evaluation necessitate further validation in larger, more controlled settings.

##### 5.1.1.3 Prognostic value of mismatch negativity latency

While the latency of MMN has shown less evidence as a prognostic marker, Wang et al. (2017) highlighted its potential, demonstrating shorter latencies in patients with MCS than those in UWS. This variation may relate to the neural generators of these responses, with MMN signals in MCS patients originating from the superior temporal and middle temporal gyri whilst, in UWS patients, they arise from the inferior and middle temporal gyri (Wang et al., 2017).

##### 5.1.1.4 Auditory discrimination using mismatch negativity

An extension of traditional MMN is the auditory discrimination paradigm, employing multivariate analysis algorithms to quantify neural responses by comparing full-montage EEG voltage topographies evoked by standard and deviant stimuli (Tzovara et al., 2013). This approach is advantageous as it requires no *a priori* prediction of amplitude and latency for the analysis window, and auditory discrimination is decoded at the individual level to accommodate inter-patient variability (Tzovara et al., 2013; Rossetti et al., 2014; Comanducci et al., 2020). Furthermore, the automated multi-channel analysis across the whole scalp mitigates artefacts and subjective bias that can lead to misinterpretation in conventional single-channel MMN (Picton et al., 2000, Tzovara et al., 2013; Rossetti et al., 2014). These paradigms have shown promise in predicting recovery of consciousness and functional outcomes. For example, Juan and colleagues demonstrated that in post-cardiac arrest patients, 86% (12 out of 14) of those showing improved auditory discrimination achieved good cerebral performance after three months, compared to only 33% of those with impaired auditory discrimination (p = 0.004) (Juan et al., 2016). Rossetti and colleagues (Rossetti et al. 2014) demonstrated similar findings, reporting a 100% PPV for awakening beyond UWS, in a cohort of 30 comatose post-HIE patients. Furthermore, patients without progression in MMN auditory discrimination over time showed significantly worse cognitive and functional outcomes (Juan et al., 2016). Testing and analysis of these paradigms were blinded to patient outcomes and clinical assessments, adding considerable strength to the data. Overall, this highlights the role of auditory discrimination MMN paradigms as effective extensions of the conventional MMN technique in the assessment of DoC.

#### 5.1.2 P300

The P300 (comprising P3a and P3b components) is a frontal and parietotemporal network potential evoked by emotionally salient stimuli within an oddball paradigm (Sutton et al., 1965, Fischer et al., 1999, Polich, 2007, Wronka et al., 2012, Morlet and Fischer, 2014, Pruvost-Robieux et al., 2022). The frontal P3a subcomponent is thought to arise from attentional processes, whereas P3b reflects subsequent perception and memory operations from parietal and temporal regions (Polich, 2007, Wronka et al., 2012). Subcomponent analysis could be prognostically useful because frontal lesions may selectively reduce P3a amplitude (Knight, 1984, Daffner, 2000) and temporoparietal lesions attenuate P3b (Knight et al., 1989). However, such relationships are inconsistent (Polich, 2007, Wronka et al., 2012) and DoC aetiologies included in the present review typically involve diffuse pathology. As with results for MMN, the presence of P300 is shown to predict favourable outcomes (Morlet et al., 2023, Wang et al., 2022). However, conflicting evidence suggests there may be no significant correlation between P300 and recovery outcomes, possibly related to methodological limitations (Portnova et al., 2023, Steppacher et al., 2013, Wang et al., 2017).

Patients with DoC who had shorter P3a latencies (p = 0.044) and larger P3a amplitude (p < 0.001) had superior prognoses after six months (Wang et al. 2022). Zhang and colleagues used the subject’s own name spoken by a familiar voice as the deviant, and either a 100 Hz tone or a pseudo-name acoustically similar to the patient’s name as the standard stimulus (Zhang et al., 2017). All five patients with P300 in both paradigms were awake within 12 months, suggesting this dual P300 stimulation paradigm may enhance sensitivities on future AEP testing. In contrast, Steppacher and colleagues reported that the presence of P300 in UWS patients did not show a significant relationship with patient outcomes (Steppacher et al., 2013). The disparities in these findings could be attributed to the differences in the structure of these studies. Specifically, the time interval until follow-up measurements displayed significant inconsistencies, with periods ranging from three months (Juan et al., 2016) to 3–12 months (Zhang et al. 2017) to approximately eight years (Steppacher et al., 2013). The significant differences in follow-up times may contribute to variability in observed recoveries and outcomes. The longer time intervals observed (Steppacher et al., 2013) allow for more extensive periods of recovery or deterioration, which could arguably offer a more accurate representation of the ability of P300 to predict long-term outcomes. Conversely, shorter follow-up durations might not capture the full extent of the patient’s recovery or the delayed onset of improvements, possibly leading to an underestimation of the prognostic ability of P300. This suggests the need for more studies with standardised follow-up intervals for better comparison between studies.

#### 5.1.3 N100

The N100 is generated by thalamocortical projections, primary auditory cortex and association areas, and signifies preservation of the auditory cortex (Hyde, 1997, Picton et al., 1999, Lehembre et al., 2012). N100 showed some capabilities to predict both positive and negative outcomes in patients with DoC, but its prognostic utility still needs further validation (Jaeger et al., 2014, Portnova et al., 2023, Rodriguez et al., 2014). An older study (Jaeger et al. 2014) found that the absence of N100 was associated with less patient autonomy in the long term, using the Functional Independence Measure. This finding was in line with reviews (Benghanem et al., 2022, Pruvost-Robieux et al., 2022) and another external study (Luaute et al., 2012). It was shown, however, that the utility of N100 may be surpassed by MMN, which is itself derived from the N100 response. In one study, out of 12 patients who displayed N100 waves, seven awakened, all of whom showed MMN waves (Rodriguez et al., 2014). Additionally, in the same study, three patients showing N100 waves with no MMN had no recovery of consciousness, suggesting that MMN is a more specific predictor of recovery. This may relate to the N100 evidencing early auditory stimulus detection, whereas MMN represents a higher function and requires additional neural processes.

#### 5.1.4 N400

The N400 is evoked by stimuli with semantic violations and neural generators are widely dispersed (Duncan et al., 2009, Lepock et al., 2022). Thought to represent retrieval of conceptual information, this high-order LLAEP is proposed as an important tool for predicting recovery in DoC (Duncan et al., 2009, Steppacher et al., 2013; Commanucci et al., 2020). Indeed, in the two included studies investigating N400 in DoC, the presence of this component was associated with a favourable prognosis (Portnova et al., 2023, Steppacher et al., 2013). A study performed by Steppacher and colleagues found that patients with preserved N400 showed a greater likelihood of regaining communication (Steppacher et al., 2013). Furthermore, when compared to P300 within the same cohort, N400 showed more reliability as a predictive marker (Steppacher et al., 2013). Additionally, the presence of N400 showed preservation of semantic or emotional processing in coma patents (Portnova et al., 2023). However, due to the limited data available, more research is still needed to confirm the effectiveness of N400 as a prognostic marker for DoC patients.

### 5.2 Brainstem auditory evoked potentials

Brainstem Auditory Evoked Potentials (BAEPs) is a sequence of short-latency waves produced with click stimuli and utilised to localise lesions of the auditory nerve and brainstem (American Clinical Neurophysiology Society (ACNS), 2006, Celesia, 2013). Conversely, the absence of BAEPs was highly specific in predicting unfavourable outcomes in DoC (Gobert et al., 2018, Lim et al., 2021, Obinata et al., 2020; Rodriguez et al., 2024). Nevertheless, the prognostic utility of BAEPs remains limited by their notably low sensitivity. One article showed a 100% specificity rate of predicting unfavourable outcomes in 47 patients with absent BAEP wave V, reinforcing the ability of this measure to identify poor outcomes (Obinata et al., 2020). As a late BAEP component, wave V originates from the high pontine or lower midbrain structures, including the inferior colliculus and medial geniculate nucleus (Legatt, 2012, Simmons and Kerasidis, 2020). In further support of this, all comatose patients who subsequently awakened displayed BAEPs, yet 90% of the non-awakened patients had intact BAEPs, demonstrating that the presence of BAEP had high sensitivity but low specificity for predicting positive outcomes (Rodriguez et al., 2014). Similarly, another study (Lim et al. 2021) showed the absence of BAEPs to have a sensitivity of only 9.4% in predicting poor outcomes in coma patients. A likely reason for the low sensitivity of BAEP is that the integrity of the brainstem is usually preserved in major DoC-causing events, such as anoxia and hypoxic-ischemic injury since these aetiologies prominently occur in the cerebral cortex (Zegers de Beyl et al., 1986, Guérit et al., 1993). All four BAEP focussed articles included in our review only included comatose patients. Therefore, future studies including other categories of DoC would be needed to make more general conclusions on their value on prognostication (Gobert et al., 2018, Lim et al., 2021, Obinata et al., 2020, Rodriguez et al., 2014).

### 5.3 Middle-latency auditory evoked potentials

Middle-latency auditory evoked potentials (MLAEPs) arise 10–50 ms after the acoustic stimulus and represent thalamic activity and the earliest cortical response at the primary auditory cortex (Liegeois-Chauvel et al., 1994, Alain et al., 2013). The prognostic ability of MLAEPs to predict outcomes in DoC patients was comparable to that of BAEPs, predicting negative outcomes with low sensitivity and a moderate ability to predict positive outcomes in the two articles that focussed on them (Jaeger et al., 2014, Rodriguez et al., 2014). It appears that the prognostic capabilities of MLAEPs are comparable to those of N100s but remain inferior to MMNs. These two studies (Jaeger et al., 2014, Rodriguez et al., 2014) however, had small sample sizes and were focused solely on comatose patients, limiting their generalisability. Larger studies exploring the capabilities of MLAEP would be advantageous to clarify their true value in DoC prognostication.

### 5.4 Comparison with somatosensory evoked potentials

The traditional view that SSEPs are useful only for predicting poor outcomes may be shifting. Simple binary assessments of SSEPs as being present or absent are moving to more quantitative and multidimensional amplitude analysis, incorporating other parameters. For instance, a recent study by Huang et al. (2025) found an N20 amplitude cut-off of >1.6 µV was 100% sensitive for awakening post-cardiac arrest. However, this amplitude threshold alone delivered a specificity of only 46.7%, suggesting a high-amplitude N20 may predict a good outcome in those patients but does not guarantee it. Scarpino and colleagues (2025) included additional metrics and reported that high N20 amplitudes (>3 µV) combined with normal waveform duration and a preserved N70 component had 94% sensitivity and 100% specificity for predicting favourable outcomes. These findings suggest that incorporating intermediate-latency components such as N70 adds prognostic value beyond short-latency SSEPs alone.

In comparison, AEPs, particularly longer-latency components, were shown in the present systematic review to also have high specificity for predicting favourable outcomes. For instance, improvement in MMN auditory discrimination achieved 100% specificity in the cohort by Rossetti et al. (2014), and the presence of P300 achieved 92% specificity in Zhang et al. (2017). As multidimensional SSEP analyses develop, they may play a complementary role alongside AEPs. Whereas quantitative SSEPs are held to indicate intact afferent sensory pathways (Klingner and Witte, 2018), the more recent results from Scarpino and colleagues (2025) on post-cardiac arrest patient outcomes indicate a higher-order cortical function relevance of SSEPs as well, as has already been established for long-latency AEPs (Oppitz et al., 2015). A multimodal approach integrating both quantitative SSEP metrics and cognitive LLAEP assessment may add value in navigating prognostic uncertainty.

### 5.5 Technical considerations for clinical implementation

AEPs in the ICU can be technically challenging. Specialist practitioners and recording techniques, along with modern high-input impedance amplifiers and signal averaging, can minimise and mitigate ambient electrical and acoustic interference (Guérit, 1999, Alvarez and Rossetti, 2015, Herman et al., 2015, British Society of Audiology, 2019, British Society of Audiology, 2022). Administering neuromuscular blockers to suppress myogenic noise can further enhance the quality of AEPs in the ICU (Saito et al., 1999, Martin and Stecker, 2008, Bithal, 2014).

### 5.6 Limitations

#### 5.6.1 Sedation

Sedative drugs can influence the results of neurophysiological prognostication in DoC patients. In the 24 articles included in this review, nine studies permitted the use of sedative drugs in their methodology, often in conjunction with the use of therapeutic hypothermia, nine studies included only patients who were weaned off the drugs, and the remaining six studies did not specify sedation status of the patients (Supplementary Table S3). These inconsistencies in addressing sedation as a potential confounding variable could impact the accuracy and comparability of the results. Sedation can also mask the results of neurophysiological assessments such as MLAEP and LLAEP (Matsushita et al., 2015). While MMN is preserved in critically ill patients undergoing deep sedation, the P300 component can be completely abolished (Azabou et al., 2018). However, when compared with healthy volunteers, MMN showed a decreased amplitude in critically ill, sedated patients (Azabou et al., 2018). A possible explanation may be that general anaesthesia such as propofol or ketamine diminishes higher-order cortical activity (Uhrig et al., 2016), which may be responsible for the present but decreased amplitude of MLAEP.

However, the duration between the cessation of the medication and AEP testing had considerable variation across studies, extending from 12 h (Rodriguez et al. 2014) to at least one week (Wang et al. 2017). Since BAEP latencies may be prolonged and MLAEP amplitudes attenuated (Benghanem et al., 2022), the inconsistencies in sedation status among studies may undermine the prognostic capabilities of these AEPs.

#### 5.6.2 Duration between disorder of consciousness onset and auditory evoked potential testing

There were significant inconsistencies in the duration between the onset of DoC and the neurophysiological assessment, ranging from an average of 15.7 h to 1247 days across different included articles (Supplementary Table S3) (Rossetti et al., 2014, Meiron et al., 2021). The difference in timing for each patient in a study may cause differences in the consciousness status of the patient, as some may be closer to awakening, showing more positive results. However, due to other treatments and managements often taking priority over prognostication, performing neurophysiological testing at a fixed interval after DoC onset remains a challenge. Of the 24 included studies, 11 relied on retrospective data collection, making uniform intervals between DoC onset and AEP testing difficult.

#### 5.6.3 Other limitations

Methodological assessment via QUADAS-2 (Fig. 4) identified many reporting gaps and methodological flaws across the included studies. Half of the included studies lacked clear recruitment protocols. Additionally, five studies applied high-risk exclusions based on complex cases that interfered with AEP testing, introducing spectrum bias and limiting generalisability. Furthermore, 15 studies did not explicitly state the blinding of outcome assessors. The lack of blinding creates a risk of a ‘self-fulfilling prophecy’, especially because these cases involve decision-making on the withdrawal of life-sustaining therapy (Roest et al., 2009). Finally, nearly half the studies were rated High Risk for patient flow. Significant attrition and exclusion of patients with artefacts or early mortality results in survivor bias (e.g., Morlet et al., 2023; Tzovara et al., 2016), potentially exaggerating the index test’s clinical utility.

Many studies were limited by small sample sizes. The median number of patients in the included studies is 35 patients. Finally, the composition of patient cohorts in the studies reveals an imbalance in DoC type. Notably, there is a predominance of coma as the type of disorder of consciousness, accounting for 67.5% (1055 out of 1562) of the patients as shown in Fig. 2. As mentioned earlier, the predominant aetiologies which cause coma, such as anoxia from cardiac arrest, may spare certain structures like the brainstem (Zegers de Beyl et al., 1986), potentially resulting in stronger BAEPs than DoC caused by other aetiologies. Notably, auditory discrimination using the MMN paradigm has been demonstrated only in coma secondary to cardiac arrest. This suggests that the results may be less applicable to patients with other aetiologies of DoC.

A key limitation of this review was the challenge of assessing the prognostic value of AEPs by aetiology. Although the majority of the included studies reported the aggregate prevalence of aetiologies and outcomes in their cohorts, they did not provide individual patient data linking specific aetiologies to AEP responses and clinical outcomes. This data gap precluded cross-tabulation required to calculate sensitivity and specificity for specific aetiological subgroups. To clarify the influence of aetiology of DoC on the prognostic value of AEPs, future research should prioritise the reporting of individual participant data. This lack of standardisation also extends to outcome measures as well. The heterogeneity of endpoints across studies, ranging from short-term awakening at discharge to long-term functional recovery, limits the ability to directly compare prognostic accuracy across cohorts.

### 5.7 Looking forward

Future research should aim to involve larger, prospective cohorts and standardised testing protocols to enhance our understanding of the prognostic potential of AEPs in DoC patients. Developing full montage EEG paradigms and multimodal prognostic strategies incorporating different AEPs will likely provide a more reliable way to prognosticate DoC patients, given the advantages demonstrated by BAEPs, MLAEPs, and LLAEPs. Additionally, visual interpretation of AEPs presents challenges, due to factors like the unfavourable signal-to-noise ratio in intensive care unit environments and inter-observer variability (Steppacher et al., 2013). Therefore, automated AEP detection paradigms may help reduce the subjectivity and inaccuracies caused by visual interpretation. Moreover, the prospect of machine learning to evaluate AEPs was explored in Floyrac et al. (2023): in 29 comatose patients, 83% sensitivity and 90% specificity were achieved in predicting patients with good versus bad neurological outcomes, suggesting a significant benefit of further, larger studies. Though currently requiring non-standard equipment and expertise, these emerging objective techniques of quantifying AEPs open avenues for more research and testing towards establishing AEPs as standard diagnostic tools worthy of integration into clinical routine.

## 6 Conclusion

We have shown that AEPs have increasingly strong and new evidence for use as prognostic tools for coma, UWS, and MCS patients. Based on current evidence, MMN paradigms can support the prediction of positive outcomes; they may also have value in indicating likely unfavourable outcomes when they are absent in single-channel recordings and when auditory discrimination using MMN is lost. Additionally, though to a lesser extent, P300 shows promise in predicting outcomes and distinguishing between the different states of consciousness. While the absence of BAEPs and MLAEPs demonstrated high specificity in predicting unfavourable outcomes, their utility remains limited due to their low sensitivity.

Existing guidelines published before the more recent articles we analysed may benefit from further development to incorporate such updated results. By advancing our understanding of AEPs and integrating them more fully with other neurophysiological techniques and standard bedside assessments, improvements may be made to aid the decision-making process for both clinicians and families facing the difficult decision of whether or not to withdraw life support in patients with DoC.

## Contributors

Panithi Khusakul conceived and designed the analysis, contributed to the analysis, conducted the study search and selection, performed full-text screening, collected the data, performed the analysis, and wrote the manuscript.

Ioan Valnarov-Boulter, Aden Noronha, and Wanapas Wachiradejkul contributed to data collection, study search, study selection, and full-text screening.

Tim Young conceived and designed the analysis, contributed to the analysis and contributed to writing and editing the manuscript.

Blake Hale contributed to the analysis and contributed to writing and editing the manuscript.

All authors have materially participated in the preparation of this manuscript.

All authors have reviewed and approved the final manuscript.

The authors declare that they have no known competing interests that could have appeared to influence the work reported in this paper.

Ethical approval was not considered necessary for the purposes of this paper.

## Data Availability

All data generated during this study are included in this article and its supplementary files.

This article was based originally on a dissertation by the lead author (PK) as part of a completed undergraduate degree iBSc in Clinical Neurology and Brain Sciences at the Queen Square Institute of Neurology, UCL, London, UK, 2024. Another co-author (TMY) was the original dissertation supervisor.

## Funding

This research received no specific grant from any funding agency in the public, commercial, or non-profit sectors.

## Declaration of competing interest

The authors declare that they have no known competing financial interests or personal relationships that could have appeared to influence the work reported in this paper.
