# Supplementary material for: Prognostic value of auditory evoked potentials in disorders of consciousness: a systematic literature review
Source: Clin Neurophysiol Pract. 2026 Jan 30;11:72–85. doi: 10.1016/j.cnp.2026.01.005 (PMC12890848; doi:10.1016/j.cnp.2026.01.005)
Supplement: Supplementary Data 2 [file mmc2.docx]

| **Study Citation** | **Reason for Exclusion** |
| --- | --- |
| **De Salvo et al. (2015)** ‘Neurophysiological assessment for evaluating residual cognition in vegetative and minimally conscious state patients: a pilot study’, Functional Neurology, 30(4), pp. 237–244. | **Ineligible Index Test:** Although the study measured N100, N200, and P300, the study assessed these components strictly as event-related potentials using visual neurosensory stimulation, rather than auditory-evoked potentials. |
| **Ling et al. (2023)** ‘Cortical responses to auditory stimulation predict the prognosis of patients with disorders of consciousness’, Clinical Neurophysiology, 153, pp. 11–20. | **Ineligible Index Test:** The study utilised frequency spectrum analysis to measure auditory-induced changes in power spectral density within delta and theta bands, rather than analysing time-locked auditory-evoked potential waveforms. |
| **Maia et al. (2013)** ‘Predicting outcome after cardiopulmonary arrest in therapeutic hypothermia patients: clinical, electrophysiological and imaging prognosticators’, Acta Médica Portuguesa, 26(2), pp. 93–97. | **Insufficient Index Test Reporting:** The study did not define the specific auditory evoked potential components or latencies analysed, reporting results as only binary (present/absent AEP). Additionally, not enough information and synthesis was given for these auditory-evoked potentials to include the study. |
| **Sokoliuk, R., et al. (2021)** ‘Covert speech comprehension predicts recovery from acute unresponsive states’, *Annals of Neurology*, 89(4), pp. 646–656. | **Ineligible Index Test:** The study assessed cortical tracking of speech envelopes using inter-trial phase coherence and neural entrainment, rather than analysing time-locked auditory evoked-potential components. |

**Supplementary Table S1:** List of Excluded Studies with Reasons For Exclusion at the Full-text Screening Stage, *This table details the citations and specific reasons for excluding studies at the full-text screening stage, such as ineligible index tests or insufficient data*
