# Supplementary material for: Prognostic value of auditory evoked potentials in disorders of consciousness: a systematic literature review
Source: Clin Neurophysiol Pract. 2026 Jan 30;11:72–85. doi: 10.1016/j.cnp.2026.01.005 (PMC12890848; doi:10.1016/j.cnp.2026.01.005)
Supplement: Supplementary Data 3 [file mmc3.docx]

| **Author & Year** | **AEP Modalities Studied** | **Country/**  **Design** | **N** | **DoC Type** | **Aetiology** | **Mean Age / Sex (Reported in Outcome Groups in Some Studies)** |
| --- | --- | --- | --- | --- | --- | --- |
| 1. Chen et al., 2020 | 40-Hz Auditory Steady-State Response | China  Prospective study | 32 | Coma (n = 32) | TBI (n = 13)  Stroke (n = 14)  Cardiac arrest (n = 3)  Others (n = 2) | - Unfavourable outcome after six months (n = 25) = 60.60 years ± 14.92 (SD), Female = 40.0% (n = 10) - Favourable outcome after six months (n = 7) = 53.29 years ± 13.76 (SD), Female = 14.3% (n = 1) |
| 1. Floyrac et al., 2023 | MMN | France  Retrospective study | 29 | Coma (n = 29) | Cardiac arrest (n = 29) | - Unfavourable neurological outcome at three to six months (n = 23) = 60 years ± 16 (SD), Female = 15.0% (n = 3) - Good neurological outcome at three to six months (n = 6) = 47.5 years ± 16 (SD), Female = 16.7% (n = 1) |
| 1. Gobert et al., 2018 | BAEP | France  Retrospective study | 7 | Coma (n =7) | Subarachnoid haemorrhage (n = 7) | *Unspecified* |
| 1. Jaeger et al., 2014 | MLAEP  N100  MMN  P300 | France  Retrospective study | 18 | Coma (n = 18) | TBI (n = 18) | 37.3 years ± 19.3 (SD), (Sex unspecified) |
| 1. Juan et al., 2016 | Auditory discrimination using MMN | Switzerland  Prospective study | 32 | Coma (n = 32) | Anoxia (n = 32) | 56 years ± 14 (SD), Female = 28.1% (n = 9) |
| 1. Levi-Strauss et al., 2023 | MMN  P300 | France  Retrospective study | 38 | Unspecified DoC | Cardiac arrest (n = 38) | Unspecified |
| 1. Lim et al., 2021 | BAEP  P100 | Korea  Retrospective study | 185 | Coma (n = 185) | Cardiac arrest (n = 185) | - Good neurological outcome at six months = 50.7 years ± 16.3 (SD), Female = 21.2% (n = 7) - Unfavourable neurological outcome at six months = 55.4 years ± 16.7 (SD), Female = 32.2% (n = 49) |
| 1. Liu et al., 2021 | MMN | China  Prospective study | 113 | Coma (n = 113) | Stroke (n = 65)  HIE from cardiac arrest (n = 28)  Intracranial infection (n = 6)  Unspecified (n = 14) | - Awakened at 3 months (n = 59) = 57.9 years ± 16.2 (SD), Female = 47.7% (n = 27) - Non-awakened at 3 months (n = 54) = 64.9 years ± 16.9 (SD), Female = 37.0% (n = 20) |
| 1. Meiron et al., 2021 | MMN | Israel  Prospective study | 10 | UWS (n = 9), MCS (n = 1) | Anoxia (n = 6)  Others (n = 4) | 61.9 years ± 9.1 (SD), Female = 10% (n = 1) |
| 1. Morgalla et al., 2014 | BAEPs | Germany  Retrospective study | 100 | Coma (n = 100) | TBI (n = 100) | 43.8 years, Female = 34% (n = 34) |
| 1. Morlet et al., 2023 | Paradigm using P300 and N200 | France  Retrospective study | 68 | Coma (n = 37), UWS (n = 17), MCS (n = 14) | Anoxia (n = 19)  Brain trauma (n = 22)  Stroke (n = 22)  Others (n = 5) | - Coma = 53 years ± 4 (SD), Female = 46% (n = 17) - UWS = 45 years ± 21 (SD), Female = 35% (n = 6) - MCS = 40.8 years ± 23.8 (SD), Female = 28.6% (n = 4) |
| 1. Obinata et al., 2020 | BAEP | Japan  Retrospective study | 124 | Coma (n = 124) | *C*ardiac arrest (n = 124) | - Favourable neurological outcomes (n = 16) = 63.5 years, Female = 25% (n = 4) - Unfavourable neurological outcomes (n = 108) = 73.0 years, Female = 38% (n = 41) |
| 1. Perez et al., 2021 | Auditory Event-Related ‘Global Effect’ | France  Prospective study | 309 | UWS (n = 138), MCS (n = 171) | TBI (n = 70)  Anoxia (n = 101)  Others (n = 138) | - 47.6% younger than 45 years old, - Female = 34% (n = 101) |
| 1. Pfeiffer et al., 2017 | Auditory discrimination using MMN | Switzerland  Prospective study | 60 | Coma (n = 60) | Cardiac arrest (n = 60) | - 67 years ± 12 (SD) - Females gender in survivor group = 23.5% (n = 8), - Female in non-survivor group = 34.6% (n = 9) |
| 1. Portnova et al., 2023 | P100  N100  P200  N200  P300  N400 | Russia  Prospective study | 24 | Coma (n = 24) | TBI (n = 24) | - Negative Outcome at 3-4 months (n = 13) = 38.14 years ± 15.87 (SD) - Good Outcome at 3-4 months (n = 11) = 39.00 years ± 12.61 (SD)   Total females = 20.83% (n = 5) |
| 1. Rodriguez et al., 2014 | BAEPs  MLAEPs (Pa wave)  N100  MMN | Canada  Prospective study | 17 | Coma (n = 17) | *C*ardiac arrest (n = 12) Other causes (n = 5) | - Awaken (n = 7) = 66 years ± 11 (SD), Female = 29% (n = 2) - Non-awakened (n = 10) = 66 years ± 14 (SD), Female = 20% (n = 2) |
| 1. Rossetti et al., 2014 | Auditory discrimination using MMN | Switzerland  Prospective study | 30 | Coma (n = 30) | HIE (n = 30) | - Alive at 3 months (n = 18) = 61.8 years ± 12.5 (SD), Female = 39% (n = 7) - Dead at 3 months (n = 12) = 61.7 years ± 13.5 (SD), Female = 25% (n = 3) |
| 1. Steppacher et al., 2013 | P300  N400 | Germany  Retrospective study | 92 | UWS (n = 53), MCS (n = 39) | TBI = 43  Hypoxia = 25  Others = 24 | - UWS = 44.5 years ± 14.5 (SD), Female = 41.0% (n = 16) - MCS = 45.0 years ± 16.9 (SD), Female = 22.6% (n = 12) |
| 1. Tzovara et al., 2013 | Auditory discrimination using MMN | Switzerland  Prospective study | 30 | Coma (n = 30) | *C*ardiac arrest (n = 30) | - Improvement in decoding performance = 59 years ± 5 (SD) - Drop in decoding performance = 63 years ± 3 (SD) - Total females = 33.3% (n = 10) |
| 1. Tzovara et al., 2016 | Auditory discrimination using MMN | Switzerland  Prospective study | 94 | Coma (n = 94) | *C*ardiac arrest (n = 75) Unspecified (n = 3)  Other (n = 16) | Survivors group based on increase/decrease from therapeutic hypothermia to normothermia:   - Increase (n = 27) 63 years ± 2 (SD) - Decrease (n = 29) = 60 years ± 3 (SD)   Unfavourable outcome group based on increase/decrease in decoding performance from therapeutic hypothermia to normothermia:   - Increase (n = 6): 67 years ± 5 (SD) - Decrease (n = 32): 63 years ± 3 (SD)   Females out of 101 patients = 25.7% (n = 26) |
| 1. Wang et al., 2017 | MMN  P300 | China  Prospective study | 11 | UWS (n = 6), MCS (n = 5) | TBI (n = 2)  Cerebral Infarction (n = 1)  HIE (n = 2)  Brainstem Infarction (n = 1)  Encephalorrhagia (n = 5) | - UWS group = 41 years ± 8.33 (SD), Female = 33.3% (n = 2) - MCS group = 44 years ± 5.2 (SD), Female = 20.0% (n = 1) |
| 1. Wang et al., 2022 | MMN  P300 | China  Retrospective study | 68 | Unspecified DoC | TBI (n = 47)  Subarachnoid haemorrhage (n = 8)  Intracerebral haemorrhage (n = 8)  Others (n = 5) | 51 years ± 16 (SD), Female = 22% (n = 15) |
| 1. Zhang et al., 2017 | P300 | China  Prospective study | 18 | Coma (n = 2), UWS (n = 9), MCS (n = 7) | Anoxia (n = 3)  Intracranial haemorrhage (n = 7)  TBI (n = 8) | 43.7 years ± 13.5 (SD), Female = 33.3% (n = 6) |
| 1. Zhou et al., 2021 | MMN | China  Retrospective study | 53 | Coma (n = 53) | TBI (n = 22)  Cerebrovascular diseases (n = 16)  Intracranial tumours (n = 5)  Unspecified (n = 10) | - Awake after six months = 48.3 years ± 15.36 (SD), Female = 18.9% (n = 7) - Non-awake after six months = 55.1 years ± 12.75 (SD), Female = 31.25% (n = 5) |

**Supplementary Table S2: Study and Patient Characteristics of Included Studies.** *This table summarises study type, location and patient demographics for each included study. Mean age and sex distribution are reported for the total cohort or stratified by outcome groups as reported by each study.*

Abbreviations: AEP = Auditory Evoked Potential; DoC = Disorder of Consciousness; SD = Standard Deviation; TBI = Traumatic Brain Injury
