# Supplementary material for: Prognostic value of auditory evoked potentials in disorders of consciousness: a systematic literature review
Source: Clin Neurophysiol Pract. 2026 Jan 30;11:72–85. doi: 10.1016/j.cnp.2026.01.005 (PMC12890848; doi:10.1016/j.cnp.2026.01.005)
Supplement: Supplementary Data 4 [file mmc4.docx]

| **Author & Year** | **Sedation and Use of Targeted Temperature Management** | **Measure of Outcome and Follow-up** | **Duration Between DoC Onset and AEP Testing** |
| --- | --- | --- | --- |
| 1. Chen et al., 2020 | Included patients free of sedative or narcotic drugs. | Followed up for six months face to face using GCS scores and CRS-R, and through telephone after discharge using GOS. | Mean = 6.1 days ± 3.4 (SD) |
| 1. Floyrac et al., 2023 | Sedation was present in 11 out of 29 patients (38%).  All patients were hypothermic (<35 ^o^C). | Patients were assessed with GCS, Simplified Acute Physiology Score II score.  GOSE retrospectively collected at three to six months. | Between day three and day six following admission  Average interval not specified |
| 1. Gobert et al., 2018 | Included patients free of sedative drugs. | Follow-up assessment was done at three, six, 12 and 24 months after onset using GOS. | Unspecified |
| 1. Jaeger et al., 2014 | Unspecified | Two to four years after brain injury using the GOS, Functional Independence Measure, Numeric Rating Scale, Quality of Life after Brain Injury, Evaluation of Attention and Memory Function, and Evaluation of Depression. | Mean = 9 days |
| 1. Juan et al., 2016 | Targeted temperature management at 33-34^o^C for 24 hours (except for five patients).  Sedation-analgesia   - Midazolam (0.1 mg/kg/h) - Fentanyl (1.5 μg/kg/h)   Shivering control   - Vecuronium (0.1mg/kg bolus) | Early cognitive functioning was assessed using a standardised neuropsychological battery.  Follow-up at three months to assess long-term global outcome using CPC through a structured telephone interview. | Within the first day after patient admission under active sedation, and another recording on the second day after sedation weaning.  Average interval not specified. |
| 1. Levi-Strauss et al., 2023 | Included patients were sedation-free for at least 24 hours at the time of recording.  The time between sedation interruption and assessment in each subgroup:  MMN+/P300+ group (n = 7) = 11.0 days [5.5-15.5]  MMN-/P300+ group (n = 8) = 16.0 days [12.3-21.3]  MMN-/P300- group (n = 21) = 15.0 days [9.0-21.3] | Patient status is assessed using clinical assessments, GCS scores and CRS-R scores.  Follow-up at three months assessing clinical outcome using GOSE through telephone. | Median [IQR] = 20.5 [15.8-29.3] days, min: 9, max: 45 days  Subgroups:  MMN+/P300+ group (n = 7) = 26.0 [20.0–34.0] days  MMN-/P300+ group (n = 8) = 18.5 [14.8–24.0] days  MMN-/P300- group (n = 21) = 20.5 [15.0–26.5] days |
| 1. Lim et al., 2021 | Targeted temperature management at 33^o^C for 24 hours. | CT scans were used for immediate prognostication, along with pupillary light reflex and corneal reflex. Neurological outcome at six months post-return of spontaneous circulation was evaluated using CPC. | Median time after return of spontaneous circulation [IQR]= 66.0 [48.9-90.1] hours |
| 1. Liu et al., 2021 | Included patients free of sedatives, anaesthetic agents, or neuromuscular blockade within 24 hours before evaluation or before being confirmed as brain dead. | Outcomes were measured by GOS three months after coma onset through telephone. | Mean = 11.4 days ± 13.7 (SD) min: 1 day, max: 79 days |
| 1. Meiron et al., 2021 | Unspecified | Clinical assessment using CRS-R and GCS at baseline.  Followed-up at 12 months using CRS-R and GCS. | Mean = 1247 days |
| 1. Morgalla et al., 2014 | Unspecified | Clinical outcome determined using GOS.  Followed up three years later using GOS. | AEP was assessed within the first three days after each patient’s trauma  Average interval not specified |
| 1. Morlet et al., 2023 | Included patients were free of sedatives for 48 hours before recording. | GCS was used at the time of test for the assessment of coma patients.  CRS-R scores were used to assess post-coma patients.  Clinical outcome at six months after recording using CRS-R and GOSE. | Mean in subgroups:  Coma group (n = 37): 12 days ± 8 (SD), range = 2-33 days  UWS group (n = 17): 4.5 months ± 3.3 (SD), range 1-10 months  MCS- group (n = 11): 4.8 months ± 3.0 (SD), range = 23 days - 10 months  MCS+ group (n = 3): 4 months ± 5.6 (SD), range = 21 days - 10 months |
| 1. Obinata et al., 2020 | Targeted temperature management was performed within 6 hours of cardiac arrest at 34-36 ^o^C for 48 hours.  Sedation   - Propofol - Midazolam   Analgesia   - Fentanyl   Shivering was managed with sedatives and analgesia | Outcome at discharge assessed with CPC score for neurological status. | Median [IQR] in subgroups:  Favourable outcome group  = 18.0 [14.5-30.5] hours  Unfavourable outcome group  = 16.0 [5.0-25.3] hours |
| 1. Perez et al., 2021 | Unspecified | CRS-R (French version), Structured phone interview at six months. | ≤3 months, n = 170  >3 months, n = 66  Average interval not specified |
| 1. Pfeiffer et al., 2017 | Targeted temperature management at 36 ^o^C for 24 hours  Sedation-analgesia   - Propofol (2-3 mg/kg/h) - Midazolam (0.1 mg/kg/h) and Fentanyl (1.5 μg/kg/h)   Shiver control   - Vecuronium - Rocuronium - Altracurium | Outcome measured using best functional level reached within 3 months after cardiac arrest using Full Outline of UnResponsiveness.  Follow-up using semi-structured phone interview at three months using CPC. | Within 24 hours after cardiac arrest  Average interval not specified |
| 1. Portnova et al., 2023 | Included patients free of pharmacotherapy or alcohol. | Outcomes were measured with GOSE. | Inclusion criteria: Patients with severe brain injury no more than eight days before study  Average interval not specified |
| 1. Rodriguez et al., 2014 | Included patients free of sedatives for at least 12 hours. | Outcomes were monitored using GCS and patients’ ability to demonstrate visible, oriented, and consistent motor and/or verbal response to commands (during hospitalisation). CPC was evaluated at discharge for functional status. | Median = 5 days |
| 1. Rossetti et al., 2014 | Therapeutic hypothermia at 33 ^o^C for 24 hours.  Sedation-analgesia   - Midazolam (0.1 mg/kg/hour) - Fentanyl (1.5 μg/kg/hour)   Shiver control   - Vecuronium (0.1 mg/kg boluses) | Functional outcome assessed with CPC at 3 months through a telephone interview. | Mean = 15.7 hours ± 5.3 (SD) |
| 1. Steppacher et al., 2013 | Unspecified | The cognitive function of patients was assessed using CRS.  Followed up from two to 15 years later after DoC onset through telephone using a structured interview. | Mean in subgroups:  MCS patients (n = 39) = 6.8 months ± 8.5 (SD)  UWS patients (n = 53) = 1.9 months ± 1.6 (SD) |
| 1. Tzovara et al., 2013 | Therapeutic hypothermia at 33^o^C for 24 hours.  Sedation:   - Midazolam (0.1 mg/kg/h) - Fentanyl (1.5 μg/kg/h)   Shiver Control   - Vecuronium (0.1 mg/kg boluses) | Level of consciousness was assessed using GCS during the initial 48 hours after coma onset.  Patients’ clinical outcomes at 3 months were recorded as awake/dead. | Mean time from the return of spontaneous circulation to the first EEG recording in subgroups:  Decoding improvement = 15 hours ± 2  Decoding drop = 15 hours ± 1 |
| 1. Tzovara et al., 2016 | Therapeutic hypothermia at 33^o^C for 24-hours.  Sedation (during 24-hour therapeutic hypothermia period)   - Midazolam (0.1/mg/kg/hr) - Fentanyl (1.5mg/kg/h)   Shiver control   - Vecuronium (0.1mg/kg/hr)   AEP recording was done off-sedation | Patient outcomes were assessed at 3 months through semi-structured phone interviews using CPC score. | Time from coma onset until first EEG recording were all done within 24 hours  Average interval not specified |
| 1. Wang et al., 2017 | Included patients free of sedatives for at least one week prior to study. | Outcome measured using Coma Recovery Scale-Revised (CRS-R).  Follow-up done at six months through telephone. | Inclusion Criteria: DoC duration **≥** three months  Average interval not specified |
| 1. Wang et al., 2022 | Excluded patients who were receiving continuous midazolam or sedatives other than dexmedetomidine / low dose of midazolam.  Dexmedetomidine (**≤ 0.7 mg/kg**/h): n = 45 (66.2%)  Midazolam (**≤ 0.7 mg/kg**/h): n = 9 (13.2%) | Outcome measured using GOSE.  Follow-up at six months through telephone. | Median [IQR] = 13 [9-22] days |
| 1. Zhang et al., 2017 | Unspecified | Behavioural assessment done using CRS-R, Longitudinal behavioural assessments were performed at 2, 6, and 12 months after initial event-related potential recordings face-to-face and through telephone depending on the hospitalisation status of patients. | Mean = 6.06 months ± 4.91 (SD) |
| 1. Zhou et al., 2021 | Included patients free of muscle relaxants or sedatives except for dexmedetomidine. | Assessed using GOS and followed up at 3 months after MMN examinations. | Median period of time from coma onset to MMN examination and GCS was 10 [1-27] days |

Supplementary Table S3: Sedation Usage, Measure of Outcomes, and Interval from Disorder of Consciousness Onset to Auditory Evoked Potential Recording in Each Study

Abbreviations: AEP – Auditory Evoked Potential; BAEP – Brainstem Auditory Evoked Potential; CI – Confidence Interval; CPC – Cerebral Performance Category; CRS – Coma Recovery Scale; CRS-R – Coma Recovery Scale-Revised; CT – Computed Tomography; –––; EEG – Electroencephalogram; GCS – Glasgow Coma Scale; GOSE – Glasgow Outcome Scale-Extended; GOS – Glasgow Outcome Scale; HIE – Hypoxic-Ischemic Encephalopathy; IQR – Interquartile Range; MCS – Minimally Conscious State; MLAEP – Middle-Latency Auditory Evoked Potential; MMN – Mismatch Negativity; NPV – Negative Predictive Value; PPV – Positive Predictive Value; SAH – Subarachnoid Haemorrhage; SD – Standard Deviation; TBI – Traumatic Brain Injury; UWS – Unresponsive Wakefulness Syndrome
